# Roles of NADPH oxidases in regulating redox homeostasis and pathogenesis of the poplar canker fungus *Cytospora chrysosperma*

**DOI:** 10.1007/s44154-025-00223-y

**Published:** 2025-05-08

**Authors:** Quansheng Li, Rongrong Guo, Aining Li, Yonglin Wang

**Affiliations:** State Key Laboratory of Efficient Production of Forest Resources, Beijing Key Laboratory for Forest Pest Control, College of Forestry, Beijing Forestry University, Haidian District, Beijing, 100083 China

**Keywords:** Poplar canker, *Cytospora chrysosperma*, NADPH oxidases, Redox homeostasis, Mitochondrial integrity, Pathogenicity

## Abstract

Poplar canker, caused by the fungus *Cytospora chrysosperma*, results in tremendous losses in poplar plantations in China. Although NADPH oxidases (NOXs) play important roles in the development and pathogenicity of several pathogenic fungi, their roles in *C. chrysosperma* remain unclear. In this study, we characterized three NOX genes (*CcNox1*, *CcNox2*, and *CcNoxR*) in *C. chrysosperma*. All three genes were highly upregulated during poplar branch infection, and deletion of any of them severely reduced virulence on poplar branches. Furthermore, deletion of either *CcNox1* or *CcNoxR* resulted in a significant increase in endogenous reactive oxygen species production in hyphae, enhanced influx of Ca^2+^, the disruption of redox homeostasis and compromised mitochondrial integrity. Moreover, biosynthesis and secretion of a known virulence factor oxalic acid was obviously defective and exogenous oxalic acid supplementation rescued the virulence of the mutants. Taken together, our findings reveal that NOXs play important roles in redox homeostasis, mitochondrial integrity and pathogenicity in *C. chrysosperma*.

**Supplementary Information:**

The online version contains supplementary material available at 10.1007/s44154-025-00223-y.

## Introduction

The ascomycete *Cytospora chrysosperma* (sexual form *Valsa sordida*) is a necrotrophic fungal pathogen that predominantly infects the branches and trunks of woody plants, causing enormous ecological and economic losses worldwide. Poplar trees are widely grown, and poplar plantations play important roles in wood production and eco-environmental protection and improvement. *C. chrysosperma* can easily attack and infect the bark of poplar trees through wounds and expands rapidly on the poplar stem, resulting in large necrotic canker on bark and even death of the tree (Lin et al*.*, 2024). To date, our strategies to effectively prevent and control this disease are scarce. Therefore, understanding the pathogenicity mechanisms of *C. chrysosperma* pathogenesis will facilitate to develop more effective disease management strategies.

Recently, dozens of pathogenicity-related genes, such as those encoding effectors (Zhang et al. 2019; Wang et al. 2022; Han et al. 2021) and transcription factors (Wu et al. 2018; Xiong et al. 2021; Xu et al. 2023; Xie et al. 2023), have been characterized in *C. chrysosperma* or *Valsa mali* (another causal agent of apple canker). Our previous study revealed that the oxalic acid (OA) secreted by *C. chrysosperma* plays a key role in suppressing the host defense response during the early stages of colonization (Wang and Wang 2020). Interestingly, reactive oxygen species (ROS) are recognized as important regulators affecting OA production in *Sclerotinia sclerotiorum* (Kim et al. 2011). On the other hand, reactive oxygen species (ROS) production, regulation and signaling have been shown to play important roles in host–pathogen interactions. However, the roles of endogenous ROS homeostasis in OA synthesis and their relationship between ROS balance and OA production during *C. chrysosperma-*poplar interaction remain unknown so yet*.*

Reactive oxygen species (ROS) is highly reactive, oxygen (and nitrogen) -bearing molecules, such as nitric oxide (NO), superoxide anion (O_2_^−^), hydrogen peroxide (H_2_O_2_), and hydroxyl radical (-OH) (Nauseef 2008). The generation of ROS occurs in plants as an early response to pathogens and infection results in a burst of ROS of plants (Zhao et al. 2023). ROSs were mainly produced by cytochrome P450, peroxisomes and NADPH oxidase (NOX) in mitochondria. In fungi, NOX is composed of three constituent subunits, NOXA (Nox1), NOXB (NOX2), and NOXC (NOX3) (Lambeth 2004). During cell differentiation, the NOX catalytic subunit responds to internal or external signals, and the catalytic subunits RacA and NoxR transfer electrons to the plasma membrane to form a multi-enzyme complex and convert O_2_ to O_2_^−^. O_2_^−^ can be rapidly converted to H_2_O_2_ by dismutase and diffused through the membrane as a second messenger, playing roles in the fungal cell wall, plasma membrane receptors, or the ion channel and activating internal signaling pathways (Scott et al. 2008). NOXs play different roles in hyphal growth, development, conidiation, and virulence in fungi (Ryder et al. 2013; Nordzieke et al. 2019; Wang et al. 2020; Wu et al. 2020). So far, the role of NOXs in *C. chrysosperma* has not been studied.

In this study, we focused on the roles of NOXs in the pathogenesis and OA production of *C. chrysosperma*. We showed that the expression of three NOX genes (*CcNox1*, *CcNox2*, and *CcNoxR*) was strongly unregulated during *C. chrysosperma* infection and that these three NOX genes are important for full virulence. Furthermore, our results indicate that *CcNox1* and *CcNoxR* are required for the endogenous ROS production, maintenance of mitochondrial integrity as well as OA synthesis. These findings emphasize the role of NOXs in pathogenesis by maintaining redox homeostasis in *C. chrysosperma*.

## Results

### NOXs are required for *C. chrysosperma* virulence

**Fig. 1:**
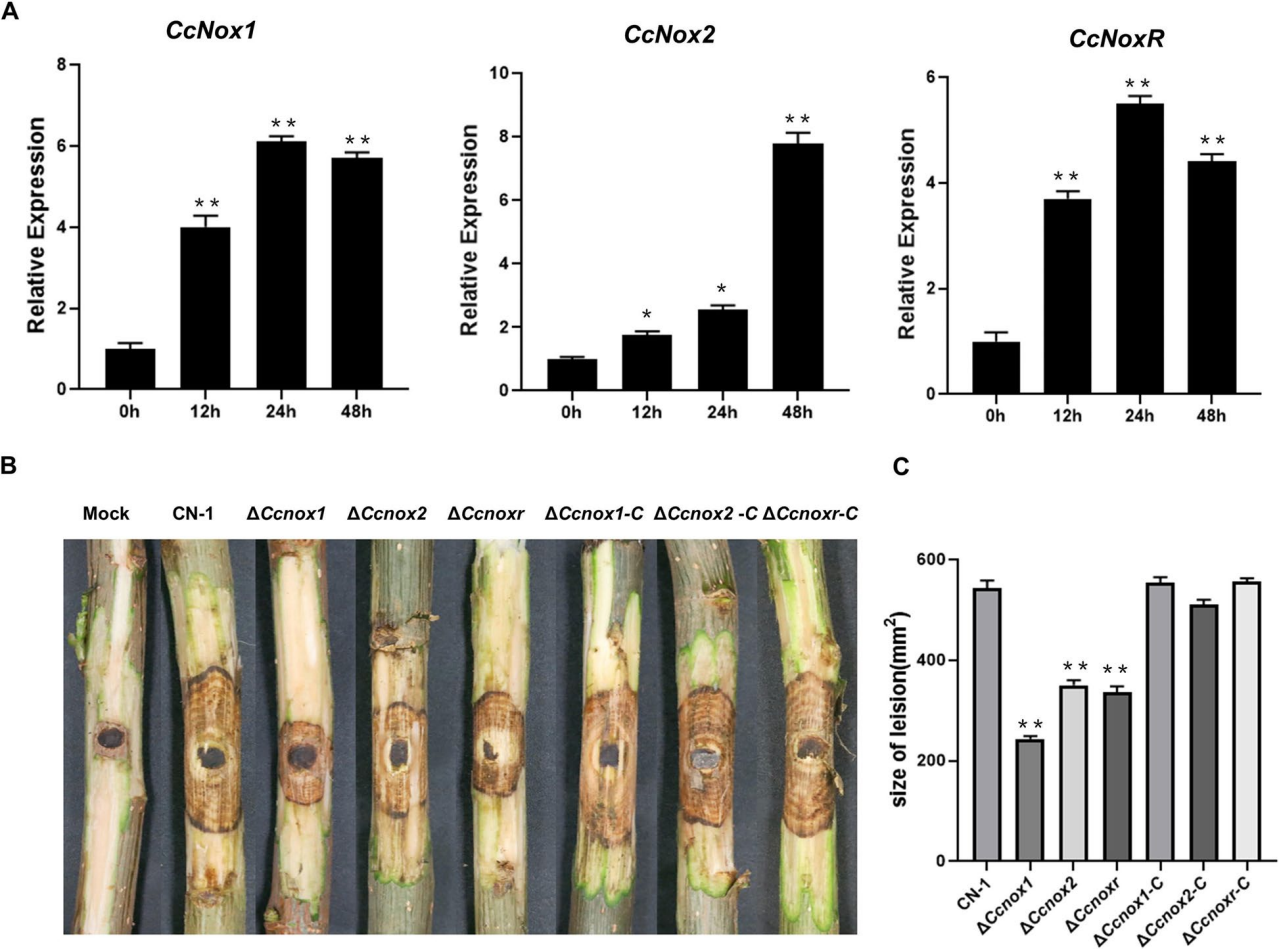
Indispensable role of NADPH oxidases in virulence of *Cytospora chrysosperma*. **A** The relative expression levels of *CcNox1*, *CcNox2*, and *CcNoxR* during *C. chrysosperma* infection was quantified at 0, 12, 24, and 48 h post inoculation (hpi), and the expression was normalized to that of the *CcAct1* gene. **B** Virulence of NOX mutants on poplar twigs. Agar plugs without fungal mycelia were used as a mock treatment. Disease symptoms were photographed 4 days post inoculation (dpi). **C** The average lesion sizes on twigs at 4 dpi. The bars indicate the standard deviations of the means of 25 individual poplar twigs. The experiment consisted of three biological replicates. The data are presented as the means ± SDs and asterisks represent significant differences (**p < 0.01)

Three homologous sequences of NOXs in the *C. chrysosperma* genome were obtained by Blastp using the NADPH oxidases protein sequences of *M. grisea* (GenBank: ABS01491.1; ABS01490.1; MGG_05280) as a template. They were named as CcNox1 (GME_2965g), CcNox2 (GME_9502g) and CcNoxR (GME_6327g). Protein domain analysis shows that CcNOX1 and CcNOX2 contain consensus motifs containing NADPH binding domains, Ferredoxin reductase-type FAD binding domain, heme binding motifs, and multiple transmembrane domains (Fig. S1A). CcNOXR contain TPR12 and PB1 domain (Fig. S1A). These findings are consistent with those reported in other fungal species (Kim et al. 2011; Mu et al. 2013; Segmüller et al. 2008). The expression levels of these three genes during *C. chrysosperma* infection compared with hyphae were examined using qRT-PCR. *CcNox1* and *CcNoxR* were both significantly upregulated in the early infection stage, and their expression peaked at 24 h post inoculation (hpi), while the expression of *CcNox2* began to increase significantly at 48 hpi (Fig. 1A).

To determine whether these three NOXs are required for *C. chrysosperma* virulence, deletion mutant strains (Δ*Ccnox1*, Δ*Ccnox2*, and Δ*Ccnoxr*) and complemented strains (Δ*Ccnox1-C,* Δ*Ccnox2-C,* and Δ*Ccnoxr-C*) were generated, respectively (Fig. S1B-D). We then examined the pathogenicity of the strains on poplar branches by inoculation with mycelial plugs of CN-1, deletion mutants, and supplemented strains. Compared with CN-1, the deletion of each NOX led to a significant decrease in virulence. The mutants *∆Ccnox1*, *∆Ccnox2*, *∆Ccnoxr* produced lesions with limited sizes on the branches, while CN-1 produced typical canker symptom at 4 dpi (Fig. 1B-C). These results suggest that NOXs play important roles in virulence of *C. chrysosperma*.

### CcNox1 and CcNoxR are involved in mycelial growth, conidiation, and resistance to oxidative stress

**Fig. 2:**
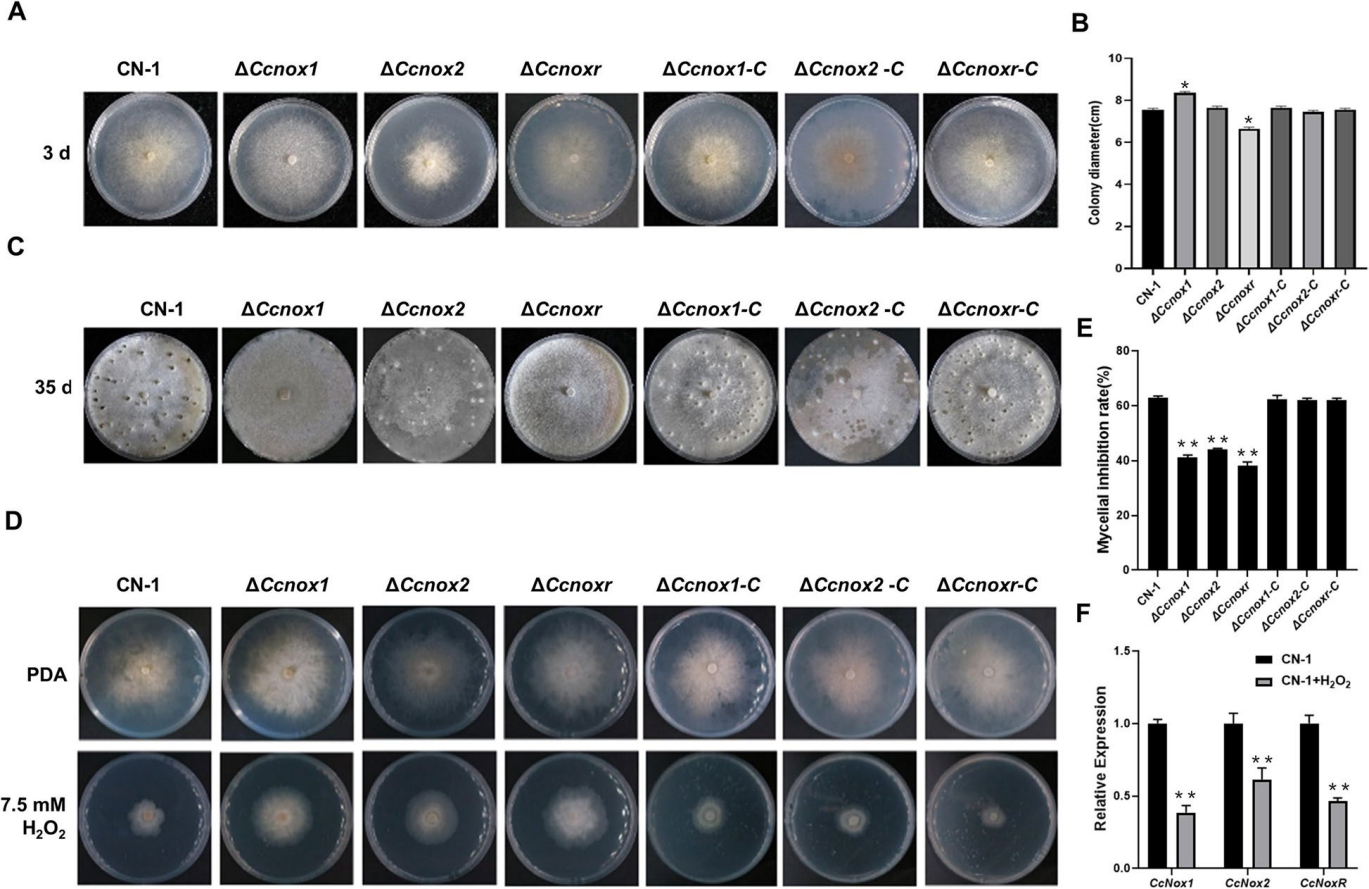
Vegetative growth, conidiation, and resistance to oxidative stress of the *∆Ccnox1*, *∆Ccnox2*, and *∆Ccnoxr* strains. **A** Colony morphology of CN-1, NOXs mutant, and complemented strains after they had grown on PDA media for 3 days at 25 °C. **B** Diameters of the colonies of the strains on PDA media. **C** Observations of the pycnidia at 35 dpi on PDA. **D** Growth phenotype on PDA media supplemented with or without H_2_O_2_ for 3 days at 25 °C. **E** Statistical analysis on the inhibition rate of all strains under H_2_O_2_ stress. **F** Expression levels of *CcNox1*, *CcNox2*, and *CcNoxR* in CN-1 in the presence or absence of hydrogen peroxide. The experiment consisted of three biological replicates. The data are presented as the means ± SDs and asterisks represent significant differences (**p < 0.01, *p < 0.05)

Compared with that of CN-1, mycelial growth of the Δ*Ccnox1* strain was slightly increased on PDA plates, while that of the Δ*Ccnoxr* strain significantly decreased, and there was no significant difference in the growth of the *∆Ccnox2* strain (Fig. 2A-B). Moreover, conidiation was completely abolished in the ∆*Ccnox1* and ∆*Ccnoxr* strains but not in the ∆*Ccnox2* strain (Fig. 2C). These results demonstrated that *CcNox1* and *CcNoxR* are important for hyphal growth and conidiation in *C. chrysosperma*.

To determine whether these NOXs respond to oxidative stress, we performed a sensitivity assay with the mutants under oxidative conditions. After the addition of 7.5 mM hydrogen peroxide (H_2_O_2_), the hyphal inhibition rate of the mutants was significantly lower than that of CN-1 (Fig. 2D-E). We further examined the expression levels of *CcNox1*, *CcNox2*, and C*cNoxR* in CN-1 under the treatment of oxidative stress and found that expression levels of *CcNox1*, *CcNox2,* and *CcNoxR* were significantly downregulated (Fig. 2F).

### Deletion of either CcNox1 or CcNoxR results in increased endogenous ROS levels and high expression of CcNox2

**Fig. 3:**
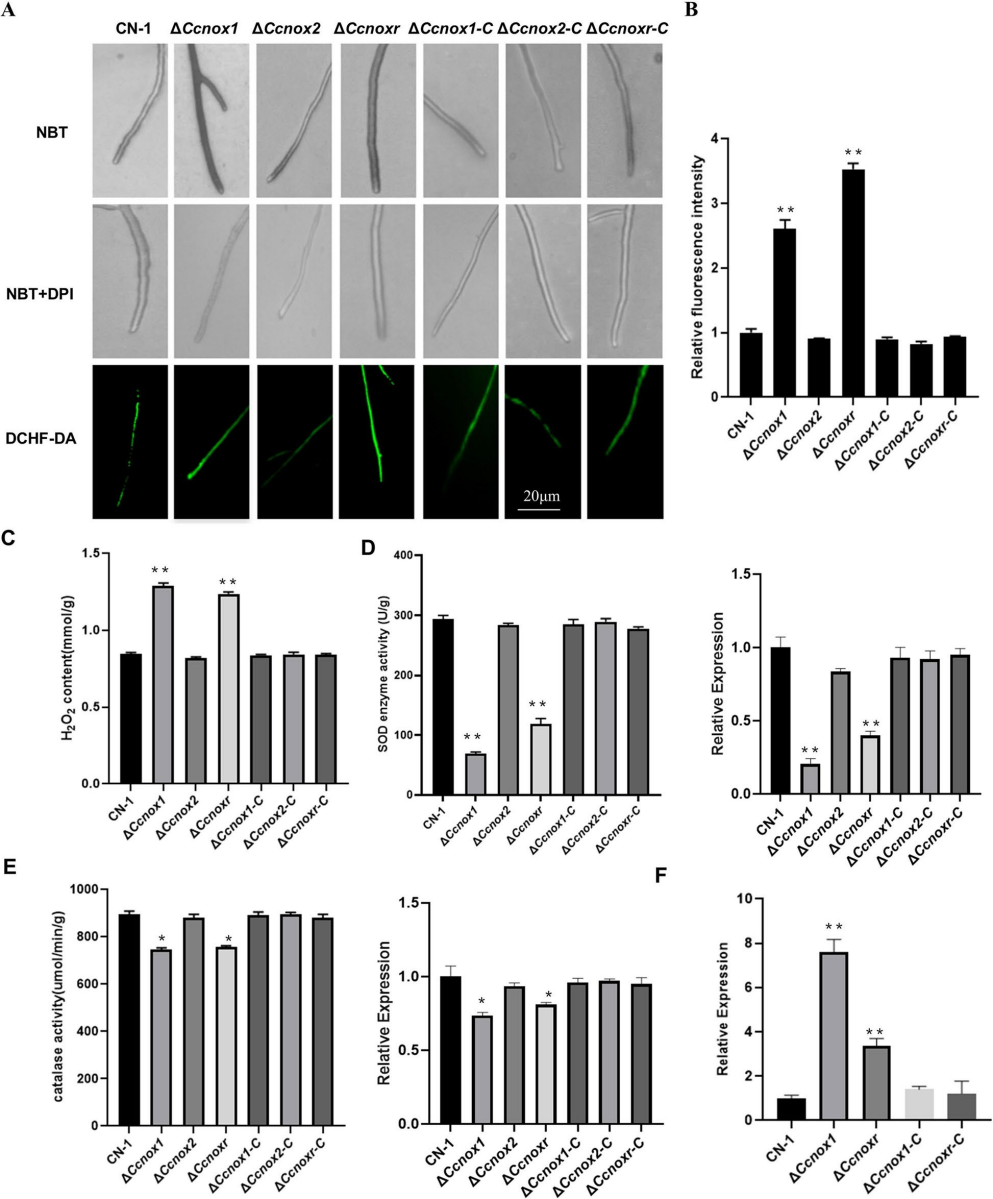
Deletion of either *CcNox1* or *CcNoxR* increased ROS accumulation. **A** The hyphae of all strains were immersed in NBT staining solution for 2 h in the presence or absence of 20 μM DPI before observation under a light microscope. The fluorescent probe DCFH-DA was used to stain the mycelia, and the accumulation of reactive oxygen species was observed under a universal fluorescence microscope. Bar: 20 μm. **B** Fluorescence intensity analysis of DCFH-DA-stained mycelium using ImageJ software.** C **Quantification of intracellular H_2_O_2_ production**.** Mycelia were cultured in PDB for 2 days and then ground with liquid nitrogen to extract H_2_O_2_ for determination.** D** Determination of SOD enzyme activity and the expression of *CcSod1* in the different strains.** E** Determination of catalase activity and the expression of *CcCat1* in the different strains. **F** The relative expression level of *CcNox2* in the *CcNox1* or *CcNoxR* strain. Error bars represent the standard deviations based on three independent replicates. The experiment consisted of three biological replicates. The data are presented as the means ± SDs and asterisks represent significant differences (**p < 0.01, *p < 0.05)

To evaluate the ROS levels in the hyphae of the NOX mutants, NBT staining showed that the Δ*Ccnox2* mutant produced formazan precipitates similar to CN-1, while the Δ*Ccnox1* and Δ*Ccnoxr* mutants produced more superoxide anions than CN-1 (Fig. 3A). After the addition of DPI, a known NOX inhibitor, CN-1 and NOXs’ mutants did not produce superoxide (Fig. 3A). Furthermore, the fluorescence intensity of DCFH-DA in the *∆Ccnox1* and ∆*Ccnoxr* strains was higher than that in CN-1, while the DCFH-DA fluorescence intensity in the *∆Ccnox2* strain was comparable to CN-1 (Fig. 3A-B).

To further explore ROS production by NOXs, we detected H_2_O_2_ content in the NOX mutants. Compared with CN-1, the H_2_O_2_ content in the *∆Ccnox1* and *∆Ccnoxr* strains increased, while that in the *∆Ccnox2* strain remained unchanged (Fig. 3C). We also examined the enzymatic activities of CAT and SOD and the expression of Cat1 and Sod1 genes in mycelium. Compared with the CN-1 and complementary strains, the ∆*Ccnox1* and ∆*Ccnoxr* strains showed significantly lower SOD enzyme activities and *CcSod1* expression (Fig. 3D). In addition, CAT activity as well as the expression of CcCat1 in the mycelium of ∆*Ccnox1* and ∆*Ccnoxr* strains were also reduced (Fig. 3E). Our results suggest that deletion of *CcNox1* and *CcNoxR* affects superoxide and superoxide dismutase activities.

**Fig. 4:**
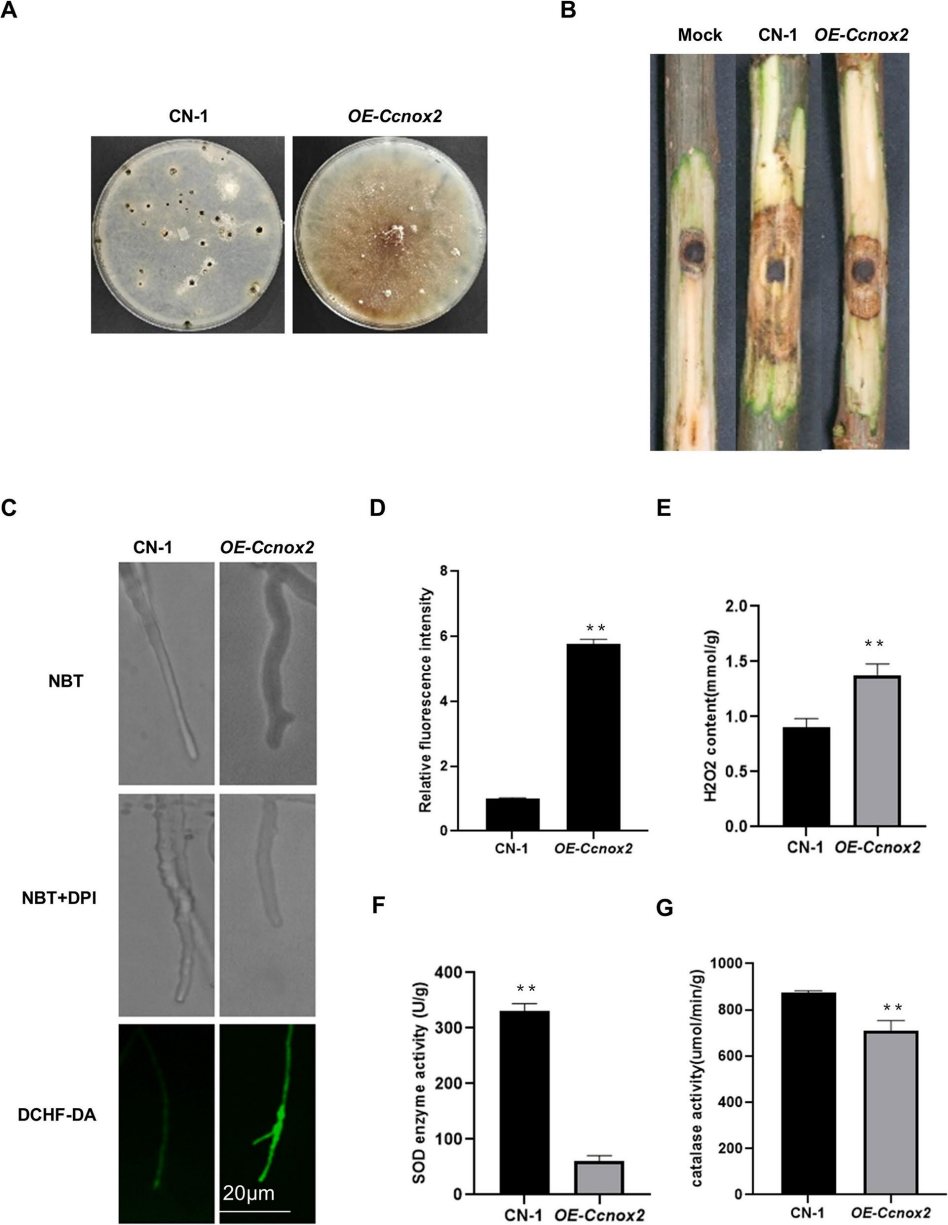
Overexpression of *CcNox2* phenocopied the Δ*Ccnox1* and Δ*Ccnoxr* strains. **A** Spore production by the *OE-Ccnox2* and CN-1 strains. **B **Virulence of *OE-Ccnox2* and CN-1 strains on poplar twigs. Agar plugs without fungal mycelia were used as a mock treatment. Disease symptoms were photographed at 4 dpi. **C **The hyphae of all strains were immersed in NBT staining solution for 2 h in the presence or absence of 20 μM DPI before observation under a light microscope (DM2500, Leica). The fluorescent probe DCFH-DA was used to stain the mycelia, and the accumulation of reactive oxygen species was observed under a universal fluorescence microscope. Bar: 20 μm. **D** Fluorescence intensity analysis of DCFH-DA-stained mycelium using ImageJ software.** E **Quantification of intracellular H_2_O_2_ production**.** Mycelia were cultured in PDB for 2 days and then ground with liquid nitrogen to extract H_2_O_2_ for determination.** F** Determination of SOD activity.** G** Determination of catalase activity. Error bars represent the standard deviations based on three independent replicates. The data are presented as the means ± SDs and asterisks represent significant differences (**p < 0.01)

Given the phenotypic similarity between ∆*Ccnox1* and ∆*Ccnoxr* and their differences from ∆*Ccnox2*, we hypothesized that there is a compensatory mechanism in which the functions of ROS are exerted by *CcNox1* and *CcNoxR* under normal conditions and that the deletion of either *CcNox1* or *CcNoxR* causes an increase in *CcNox2* expression. We measured the *CcNox2* expression levels in the ∆*Ccnox1* and ∆*Ccnoxr* mutants. As predicted, these values were 7.4-fold and 3.6-fold greater than those in CN-1 (Fig. 3F). Additionally, we generated a strain overexpressing *CcNox2* (*OE-Ccnox2*) (Fig. S1E), and found that the ROS level in the mycelia of the *OE-Ccnox2* strain significantly increased (Fig. 4C-E). We also detected the activities of CAT and SOD in the mycelia of the overexpressing strain. Compared with CN-1, the SOD and CAT activity in the *OE-Ccnox2* strain was significantly decreased (Fig. 4F-G). In addition, we observed that the OE-Ccnox2 strain was completely unable to produce conidia and exhibited significantly reduced virulence (Fig. 4A-B), which is similar to the ∆*Ccnox1* and ∆*Ccnoxr* strains. These results suggest that CcNox1 and CcNoxR play major roles in cellular ROS production in *C. chrysosperma*.

### Deletion of either CcNox1 or CcNoxR leads to Ca^2+^ influx and disrupted redox homeostasis

**Fig. 5:**
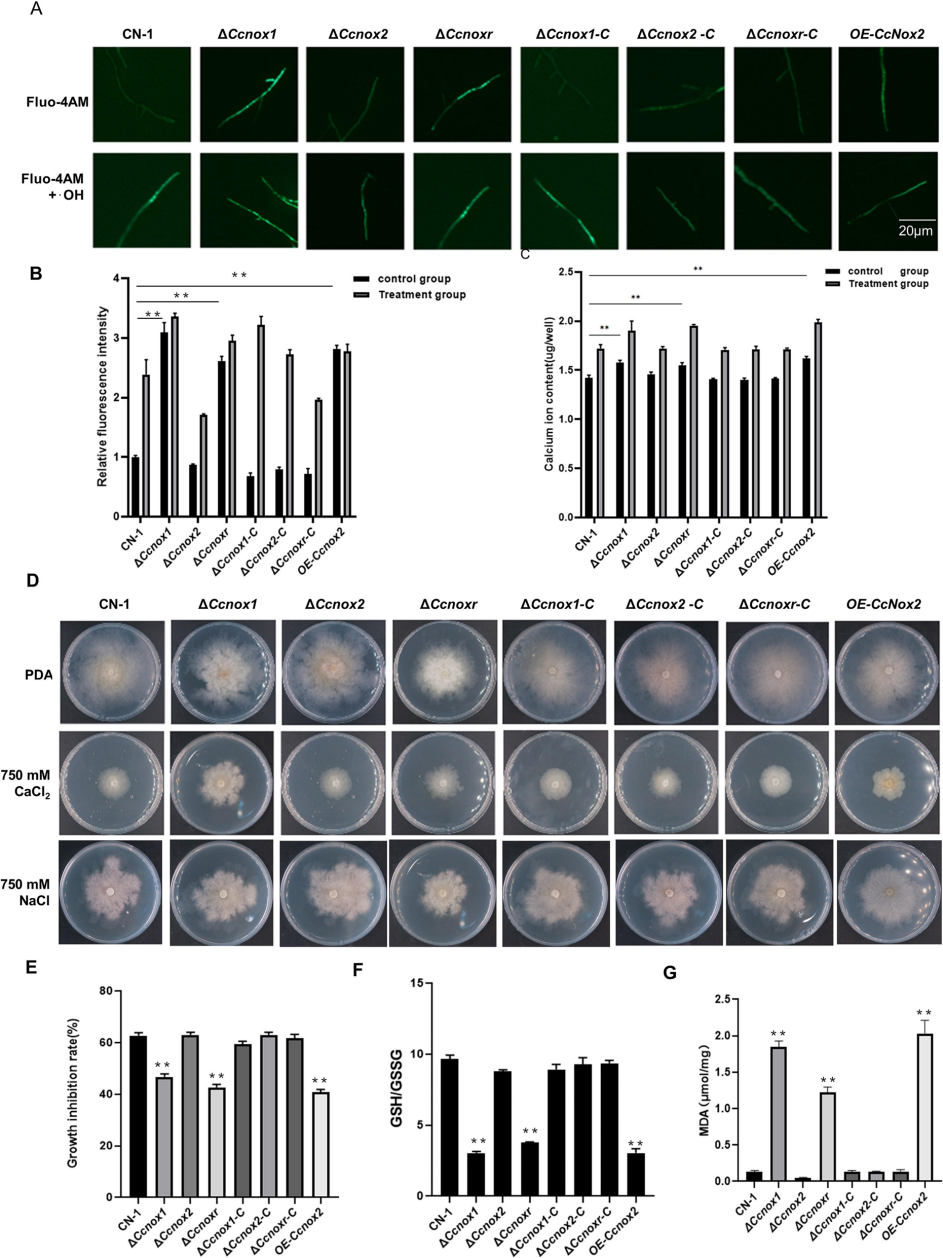
Deletion of either *CcNox1* or *CcNoxR* led to an increase in Ca^2+^ influx and the disruption of redox homeostasis. **A** The content of free calcium ions (Ca^2^⁺) in different strains was detected using the fluorescent indicator Fluo-4 AM Fluorescence. Under untreated conditions, ∆*Ccnox1*, ∆*Ccnoxr* and *OE-Ccnox2* strains exhibited higher fluorescence intensity. The fluorescence intensity of all strains was further enhanced by the introduction of hydroxyl radicals (·OH) produced by a mixture of 2 mM H₂O₂, 0.5 mM Cu^2^⁺ and 0.5 mM ascorbic acid. **B** Fluorescence intensity analysis of Fluo-4 AM-stained mycelium using ImageJ software. Significance analysis was performed for each strain in the control group. **C **The Ca.^2^⁺ content was quantified using a calcium ion detection kit in the presence and absence of -OH treatment. Significance analysis was performed for each strain in the control group. **D** In total, 750 mM CaCl_2_ was added to PDA for 3 days of culture to observe the morphology of all the strains. **E** Mycelia inhibition rates.** F** Measurement of glutathione (GSH) and oxidized glutathione (GSSG) contents. **G** Determination of MDA content. Error bars represent the standard deviations based on three independent replicates. The experiment consisted of three biological replicates. The data are presented as the means ± SDs and asterisks represent significant differences (**p < 0.01)

ROS generation by NOXs in fungi can be converted to hydroxyl radicals (·OH), mediating Ca^2+^ endocytosis in the presence of transition metals such as Cu^2+^ or Fe^2+^ (Zhao et al. 2016). Therefore, we examined the free calcium content in the mycelia and found that ∆*Ccnox1*, ∆*Ccnoxr*, and *OE-Ccnox2* had established a high Ca^2^⁺ gradient, and the application of hydroxyl radicals(·OH), produced by a mixture of 2 mM H₂O₂, 0.5 mM Cu^2^⁺, and 0.5 mM ascorbate, further increased the calcium ion concentration in all strains (Fig. 5A-C). Next, we tested the sensitivity of all strains to high concentrations of Ca^2+^ (with Na^+^ as a control). The results showed that ∆*Ccnox1*, ∆*Ccnoxr* and *OE-Ccnox2* strains significantly showed reduced growth after the addition of 750 mM CaCl_2_ to PDA compared with CN-1, whereas there was no significant difference in the growth of these strains after the addition of 750 mM NaCl (Fig. 5D-E).

When the ROS level exceeds the endogenous antioxidant capacity, disruption of the normal redox state in the cellular environment can result, inducing oxidative stress in cells. To determine whether oxidative stress occurred in the NOXs’ mutants with increased levels of ROS, we measured indicators related to oxidative stress: (1) The ratio of GSH/GSSG reflect the state of intracellular redox balance in the reduced (GSH) and oxidized (GSSG) state. The treatment of oxidative stress can lead to a decrease in GSH/GSSG levels. (2) MDA (Malondildehyde), an oxidative product whose levels can reflect the impact of oxidative stress on lipid peroxidation. The results showed that the ratios of GSH/GSSG were 0.26-fold, 0.28-fold, and 0.29-fold greater and the MDA contents were 12.75-fold, 14.25-fold, and 9.75-fold greater in the Δ*Ccnox1*, Δ*Ccnoxr,* and *OE-Ccnox2* strains than that in CN-1, respectively (Fig. 5F-G). These data suggest that CcNox1 and CcNoxR are mainly involved in maintaining Ca^2+^ and redox homeostasis.

### CcNox1 and CcNoxR are required for mitochondrial structure integrity

**Fig. 6:**
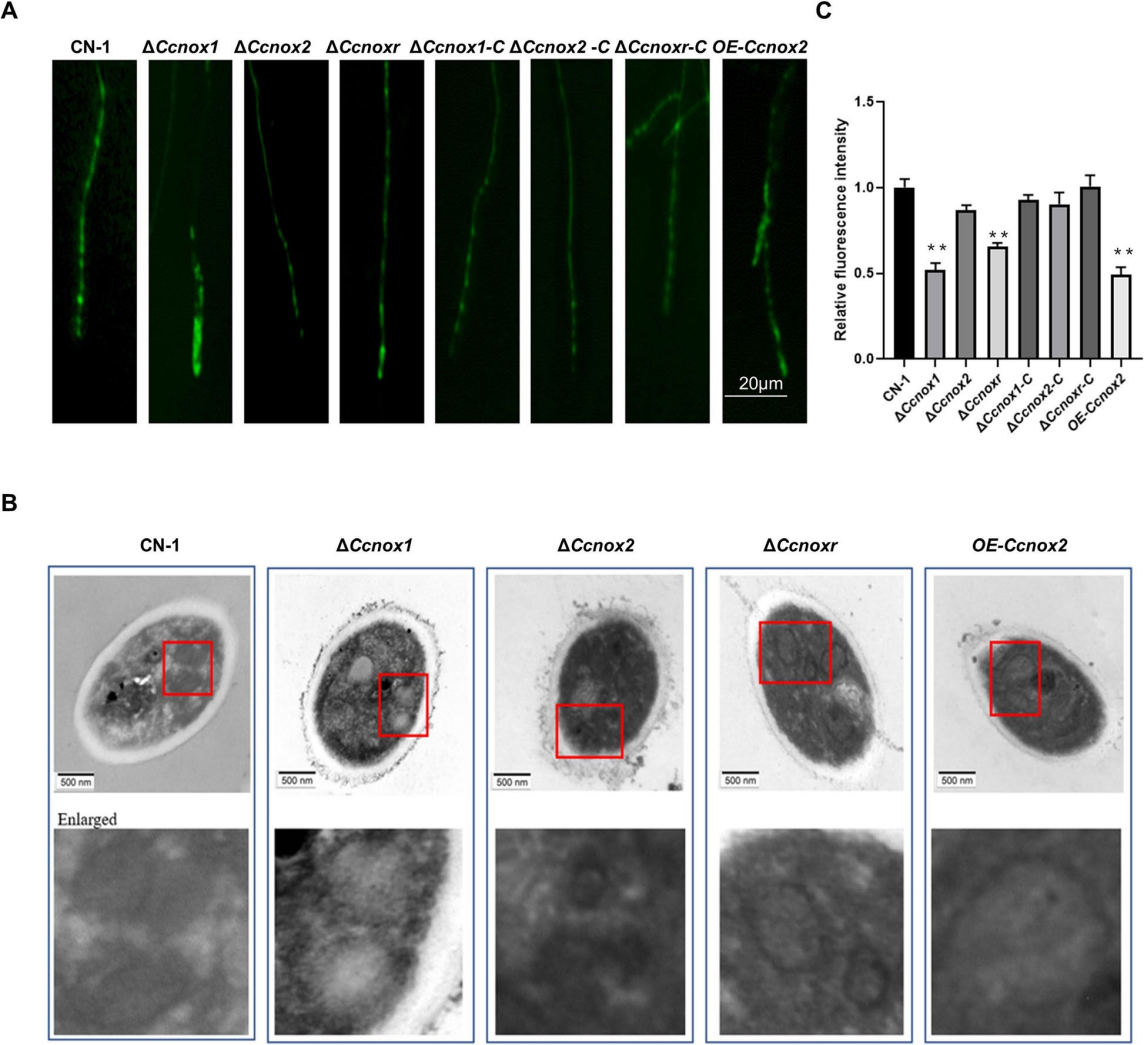
*CcNox1* and *CcNoxR* are critical for mitochondrial integrity. **A** Mito-Tracker Green, a mitochondrial probe that emits green fluorescence, was used to stain the mitochondria in mycelial cells, which were then observed using a universal fluorescence microscope. **B** After culturing the mycelium in PDB for 3 days, it was fixed with 2.5% malondialdehyde for 24 h. The mitochondria were observed using transmission electron microscopy. **C** The mitochondrial membrane potential was measured using TMRE, a tangerine cationic fluorescence probe that can penetrate the cell membrane. The change in the mitochondrial membrane potential was determined by measuring the intensity of the fluorescence signal with a fluorescence microplate analyzer. The experiment consisted of three biological replicates. The data are presented as the means ± SDs and asterisks represent significant differences (**p < 0.01)

Severe oxidative stress may result in damage to the mitochondria, which results in excessive production of ROS. Therefore, we evaluated the morphology, distribution, and membrane potential of the mitochondria and its ultrastructure was observed using transmission electron microscopy (TEM). Compared to those in CN-1, the mitochondria in the *∆Ccnox1*, *∆Ccnoxr,* and *OE-Ccnox2* strains were significantly swollen (Fig. 6A). Subsequently, Mito-Tracker Green was used to observe mitochondrial localization using TEM. Mitochondria at the tip of the mycelium had aggregated in the *∆Ccnox1*, *∆Ccnoxr,* and *OE-Ccnox2* strains, which is consistent with the localization of ROS (Fig. 6B). Then, we used TMRE, a fluorescent probe that indicates mitochondrial membrane potential, to measure the mitochondrial membrane potential. Compared with CN-1, the membrane potentials of the *∆Ccnox1* and *∆Ccnoxr* strains decreased significantly (Fig. 6C). These findings suggest that CcNox1 and CcNoxR are critical for maintaining mitochondrial integrity.

### CcNox1 and CcNoxR are involved in OA biosynthesis

**Fig. 7:**
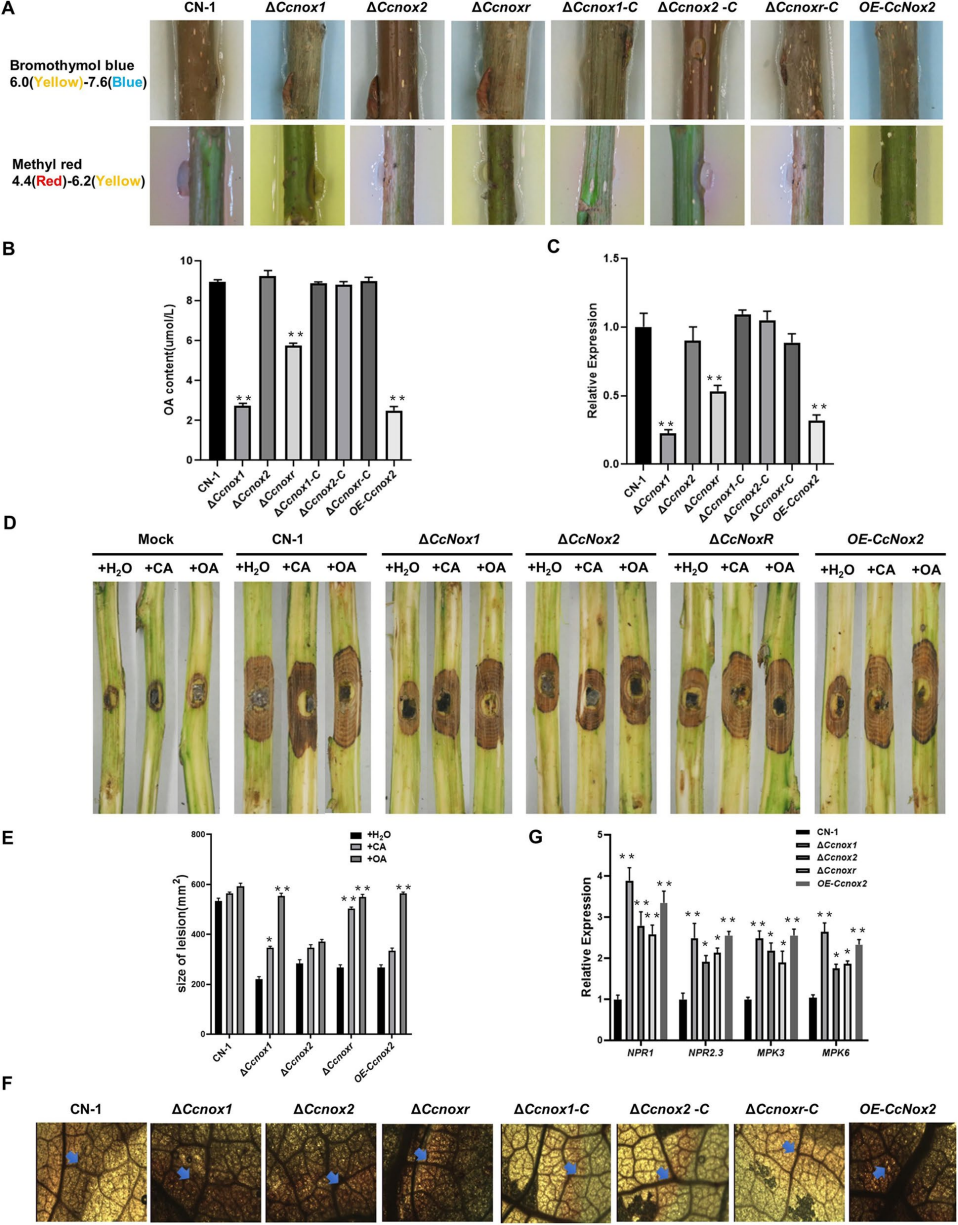
*CcNox1* and *CcNoxR* are involved in oxalic acid biosynthesis. **A** Plate acid–base indicator experiment. A representative color change was observed on different plates supplemented with methyl red and bromophenol blue. **B** Oxalic acid production in potato dextrose broth (PDB). Each strain was cultured for 3 days, and the resulting liquid culture was analyzed for oxalate accumulation. **C** Expression level of the oxalate synthase gene *CcOah*. **D** The development of diseased spots on poplar branches. OA and CA were used to treat the branches before vaccination. Each strain was inoculated with an agar plug with a diameter of 5 mm; sterile water served as a control. Photos were taken at 3 days after vaccination. **E** Quantitative analysis of lesion areas. **F **Scalded poplar leaves were inoculated with fungal strains, and after 24 h of inoculation, the detached leaves were stained with 0.01% 3,3′-diaminobenzidine (DAB) for ROS detection and decolorized with 95% alcohol. The blue arrow indicates the ROS activated by the strain. **G **Expression of defense genes (NPR1, NPR2.3, MPK3, and MPK6) in poplar trees. RNA of the poplar branches was extracted at 24 hpi for qRT‒PCR analysis. The experiment consisted of three biological replicates. The data are presented as the means ± SDs and asterisks represent significant differences (**p < 0.01, *p < 0.05)

During early infection stages, *C. chrysosperma* produced OA to promote the pathogenesis (Wang and Wang 2020). Due to the mitochondrial abnormalities of the *∆Ccnox1* and *∆Ccnoxr* strain*,* we next examined the ability of each strain to produce acids using acid–base indicators (bromophenol blue and methyl red). Notably, OA production was significantly reduced in the *∆Ccnox1, ∆Ccnoxr,* and *OE-Ccnox2* strains (Fig. 7A). Next, the OA levels in the NOXs’ mutants and CN-1 were determined at 5 dpi on poplar branches. The OA contents in the *∆Ccnox1*, *∆Ccnoxr,* and *OE-Ccnox2* strains were only approximately one-quarter of that in CN-1, while the OA content in the supplemented strain was comparable to CN-1 (Fig. 7B). We further measured relative expression levels of OA synthesis-related gene (*CcOah*) in all strains by qRT-PCR and showed that the expression level of *CcOah* was significantly decreased in the *∆Ccnox1*, *∆Ccnoxr*, and *OE-Ccnox2* strains (Fig. 7C). Furthermore, exogenous addition of CA and OA to the mutants restored the virulence defects of the *∆Ccnox1*, *∆Ccnoxr*, and *OE-Ccnox2* (Fig. 7D, E).

Since reduced OA production leads to the strongly localized production of ROS in poplar leaves (Wang and Wang 2020), the results showed that ROS production clearly increased around the infection site of the *∆Ccnox1*, *∆Ccnoxr*, and *OE-Ccnox2* strains (Fig. 7F). Subsequently, we quantified the expression levels of defense-related genes (*NPR1*, *NPR2.3*, *MPK3*, and *MPK6*) in poplar after inoculation with the strains. The results showed significant increases of these defense-related genes when inoculated with the NOXs’ mutants compared with CN-1 (Fig. 7G). The results suggest that NOXs are involved in the outcome of *C. chrysosperma*-poplar interactions.

## Discussion

Compared with detailed information on interactions between pathogen and crops, little is known about woody plant-pathogen interaction. Poplar canker fungus *C. chrysosperma* is a typical forest pathogen and the poplar canker is one of the most destructive forest diseases and is hard to control. Underlying mechanism of poplar-*C. chrysosperma* interaction will facilitate disease control of poplar canker and forest pathology. In this study, we focused on how ROS was generated by NOXs and ROS was involved in pathogenesis of *C. chrysosperma.*

The ROS generated by the NOX family serve as signaling molecules that regulate hyphal differentiation and sexual reproduction processes in various fungi. Here, we report the convergent and distinct roles of three NOXs in mycelial growth and conidiation in *C. chrysosperma*. The Δ*Ccnox1* mutant exhibited increased mycelial growth, while the Δ*Ccnoxr* strain showed reduced growth, and the Δ*Ccnox2* strain was comparable to CN-1. The impacts of NOXs on mycelial growth vary across different fungi. For instance, in *Curvularia lunata* and *Penicillium expansum*, NOXs negatively regulate mycelial growth (Wang et al. 2020; Zhang et al. 2021). Furthermore, we observed that the Δ*Ccnox1* and Δ*Ccnoxr* strain could not produce conidia, indicating that CcNox1 and CcNoxR are required for the asexual reproduction. On the contrary, increased spore formation was observed in the Δ*AanoxA* and Δ*AanoxB* strains of *Alternaria alternate* (Yang and Chung 2013). These results suggest that NOXs have various functions in filamentous fungi.

NOXs play essential roles in the resistance of fungi to adverse environments. They can promote the cellular differentiation, growth, and survival of fungi by producing ROS and enhancing resistance to oxidative stress (Ewald et al. 2017). The Δ*Ccnox1*, Δ*Ccnox2*, and Δ*Ccnoxr* mutants exhibited reduced sensitivity to H_2_O_2_. Similar findings have been documented in *P. expansum* and *A. alternata*, where Δ*noxA/B* and *ΔnoxB* displayed varying sensitivities to oxidative stress (Yang and Chung 2013; Zhang et al. 2021). However, unlike *P. expansum* and *A. alternata*, *C. chrysosperma* NOXs may not play a role in cell wall integrity.

Generally, the loss of NOX typically results in a reduction in ROS levels (Kim et al. 2011; Wang et al. 2020; Yang and Chung 2012; Morita et al. 2012; Liu et al. 2022). However, in *C. chrysosperma*, we observed an increase in ROS in the Δ*Ccnox1* or Δ*Ccnoxr* mutants, which is a phenomenon also noted in *Magnaporthe oryzae* and *Podospora anserina* (Ryder et al. 2013; Malagnac et al. 2004). When Cc*Nox1* is deleted, the expression of *CcNoxR* is slightly decreased, when *CcNoxR* is deleted, the expression of *CcNox1* is unaffected. Interestingly, our findings showed significantly upregulated expression of *CcNox2* in the Δ*Ccnox1* or Δ*Ccnoxr* mutants. Furthermore, the phenotype of the *OE-Ccnox2* strain indicated the compensatory mechanism between *CcNox1/CcNoxR* and *CcNox2*, demonstrating that *CcNox1*, *CcNoxR*, and *CcNox2* have different roles in regulating ROS in *C. chrysosperma*. When the functions of *CcNox1* or *CcNoxR* are disrupted, the production of ROS is continued via *CcNox2*, although this may cause ROS levels to exceed normal requirements. Unfortunately, we were unable to obtain a *Nox1*/*Nox2* double-knockout mutant for validation.

Exogenous oxidative stress caused by hydrogen peroxide or tert-butyl hydrogen peroxide induces the release of Ca^2+^ in yeast and then mediates the cytotoxic effects of stressors (Popa et al. 2010). In the fungus *Ganoderma lucidum*, ROS activate calcium channels and cause Ca^2+^ to flow inward. The number of mycelial branches and levels of metabolites were also related to the content of Ca^2+^ (Mu et al. 2013). In *Verticillium dahliae*, the ROS content affects the content of Ca^2+^ in the mycelia, which in turn affects the formation of invasive nails (Zhao et al. 2016). In *C. chrysosperma*, ROS can activate calcium channels on the plasma membrane and promote the influx of Ca^2+^, thus affecting the Ca^2+^ content. These results show that ROS regulate the Ca^2+^ pathway.

When the ROS level exceeds the endogenous antioxidant defense capacity, the normal redox state in the cellular environment can be disrupted, causing oxidative damage to the cells (Sharma et al. 2012). A feedforward regulatory relationship between mitochondria and NOX implies that excessive activation of NOX may increase mitochondrial ROS production, inducing oxidative stress and ultimately causing damage to the mitochondria (Dikalov 2011). However, there have been no reports of this phenomenon in fungi. Since the ratio of GSH/ GSSG reflect the intracellular redox statu (Nuhu et al. 2020) and MDA was used to reflect the cellular lipid peroxidation level (Shen et al. 2020). Here, Δ*Ccnox1* and Δ*Ccnoxr* mutants showed a significant increase in GSH/GSSG ratio and MDA content, indicating that the mutants were under severe oxidative stress. Furthermore, severe oxidative stress causes oxidative damage to the mitochondria (Zhang et al. 2023), we showed clear mitochondrial abnormalities including mitochondrial swelling and membrane potential decrease, indicating a correlation between the elevated ROS levels and mitochondrial oxidative damage in the *∆Ccnox1* and *∆Ccnoxr* mutants.

Disrupting mitochondrial function can affect the normal synthesis of downstream metabolic pathways(Martínez-Reyes et al. 2016), previous studies showed that *C. chrysosperma* requires acidic substances (such as OA) to promote infection (Wang and Wang 2020). This phenomenon was also observed for *S. sclerotiorum*, which is also a necrotrophic fungal pathogen (Rana et al. 2022). In *S. sclerotiorum*, silencing *SsNox1* leads to a decrease in OA synthesis (Kim et al. 2011). Therefore, we investigated the impact of NOXs deficiency on acid production and found that in plants infected with *∆Ccnox1*, *∆Ccnoxr,* and *OE-Ccnox2*, OA production is affected and the production of ROS is promoted, which may partly explain reduced virulence. We also determined whether this effect on OA synthesis is caused by strong oxidative stress in the mutant. H_2_O_2_ is a typical oxidant that can induce oxidative stress in the body. Therefore, we detected the OA content in fungi growing in the water of poplar branches treated with H_2_O_2_ to simulate the change in OA content under oxidative stress. The results showed that the OA content was negatively correlated with H_2_O_2_ concentration, consistent with the increase in ROS and OA content observed in ∆*Ccnox1*, ∆*Ccnoxr*, and *OE-Ccnox2*. Whether other factors associated with ROS production actually affect OA biosynthesis and the specifics of the effect need to be further investigated.

We found that *CcNox2* had a different expression pattern from *CcNox1* and *CcNoxR* and the deletion of *CcNox2* showed significant differences in iron ion uptake and sensitivity (Fig. S2A-C). Subsequent results showed that *CcNox2* affected the expression of iron carrier-related genes and reduced iron carrier synthesis (Fig. S2D-F), indicating that *CcNox2* seems to play a role in virulence by regulating iron uptake by *C. chrysosperma*, which requires further research. Since OA is an important virulence factor, especially early infection stage, we believe that CcNox1 and CcNoxR have important roles in this process, and, given the importance of CcNox2 in counteracting high iron concentrations, we investigate whether Nox1 and Nox2 play different roles at different stages of infection. We attempted to observe the morphology of the mutants at different stages of infection. Unfortunately, due to the specificity of the poplar branches infested with *C. chrysosperma* and the lack of technical means, we did not observe any differences in mycelial morphology and secretion during the different stages of infection.

## Conclusion

**Fig. 8:**
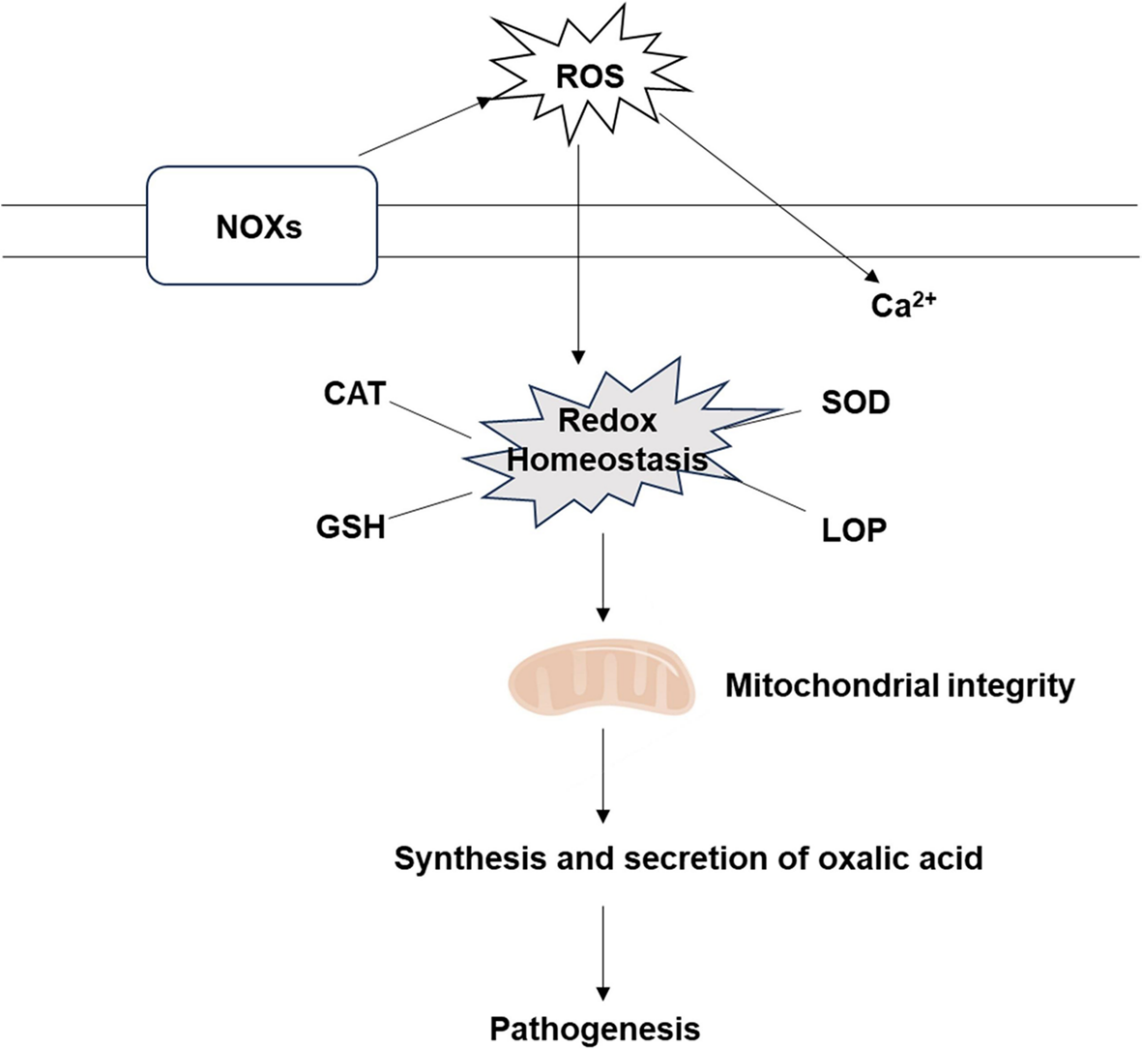
A working model of NOXs for pathogenesis in *Cytospora chrysosperma*. Under normal circumstances, two NOX enzymes (NOX1 and NOX2) may play different roles. NOX1 and NOXR play a role in the production of reactive oxygen species, while NOX2 plays an iron redox role. When NOX1 or NOXR is disrupted, it will induce overexpression of NOX2, produce excessive reactive oxygen species, and cause cells to be in a state of oxidative stress. When NOX1 or NOXR is disrupted, it will induce overexpression of NOX2, produce excessive reactive oxygen species, and cause cells to be in a state of oxidative stress. Severe oxidative stress, on the one hand, leads to lipid peroxidation, on the other hand, leads to mitochondrial abnormalities, such as mitochondrial swelling, membrane potential decline, and more mitochondrial reactive oxygen species. The abnormality of mitochondria not only disrupts the normal progress of the tricarboxylic acid cycle, leading to a decrease in oxalic acid synthesis, but also causes attenuated virulence

In summary, our studies identified the roles of NADPH oxidases in maintaining redox homeostasis, secondary metabolite production, and pathogenicity. In particular, we identified their role in maintaining fungal mitochondrial integrity for the first time (Fig. 8), providing new insights into the role of NADPH oxidases in filamentous fungi. In the future, fungicides targeting NADPH oxidase may offer potential avenues for controlling poplar canker disease.

## Materials and methods

### Strains and culture conditions

The *C. chrysosperma* wild-type strain CN-1 was isolated from *Populus alba* var*. pyramidalis* in Gansu Province, China. CN-1 and its derived mutants were inoculated in PDA media at 25 °C. PDB medium was mainly used for shaking hyphal cultures.

### Generation of targted deletion mutants, complementation and overexpression strains

The genes *CcNox1*, *CcNox2*, and *CcNoxR* were disrupted using the split marker method (Goswami 2012), respectively. The recombinant fragment was then introduced into CN-1 protoplasts via PEG-mediated genetic transformation, with the hygromycin cassette replacing the target gene. The transformants grew on PDA media supplemented with 35 μg/ml hygromycin. Mutants were screened by PCR validation of the transformants with internal and external primers (Table S1).

To obtain complements, the entire coding region plus about 2000 bp putative promoter region was amplified via PCR as complementary DNA fragments. These fragments, along with geneticin cassette, were subsequently introduced into protoplasts of the corresponding mutant simultaneously.

To obtain an overexpression strain expressing *Nox2*, the Trpc promoter was fused to the entire *Nox2* coding region using overlapping PCR, and the DNA fragments with geneticin cassettes were introduced into protoplasts of the Δ*Ccnox2* strain or CN-1. The transformants were subsequently grown and screened on PDA media supplemented with 75 μg/ml geneticin, after which the complemented and overexpression strains were verified via PCR.

### Virulence analysis

The branches and leaves of 1-year-old poplar branches from the nursery of Beijing Forestry University were used for the virulence assay. The branches were cut into 20 cm sections and washed with distilled water, then two ends of each branch were sealed with paraffin to prevent water loss. Each twig segment was firstly scalded, and a mycelial block 5 mm in diameter was obtained from the edge of the active colony by using a punch. This mycelial block was then inoculated onto the wound, wrapped, and secured with film to maintain freshness. The inoculated branches were placed in trays, sprayed daily with sterile water to maintain humidity, and incubated at 25°C in a day/night cycle. Upon onset, the lesion area was measured and photographed. At least 25 poplar branches were treated with each strain each experiment.

For exogenous application of OA or citric acid (CA), an aqueous solution of OA (100 μg/ml) or CA (100 μg/ml) was added dropwise to the scalded area of branches and leaves. Subsequently, the pretreated twigs and leaves were inoculated with mycelial plugs as described above.

### Detection of ROS levels

For superoxide detection, aspirate 0.5 ml of melted PDA medium onto a sterilized slide to make a uniform layer, wait for it to cool down and then inoculate the strain onto the slide medium, then put the slide into a petri dish and incubate it at 25 ℃ for 1–2 days in an incubator. The hyphae of all strains were immersed in 0.05% nitro blue tetrazolium (NBT) staining solution for 2 h in the presence or absence of 20 mM diphenyleneiodonium (DPI) and protect it from light. At the end of the reaction, rinsing with anhydrous ethanol was performed to terminate the reaction process. Under a light microscope, we observed ROS accumulation within the mycelium.

### Assays on activities of superoxide dismutase (SOD) and catalase (CAT)

For the fluorescence assay, mycelia were inoculated on sterile glass slides for 1–2 days, stained with 10 µM dichlorodihydrofluorescein diacetate (DCFH-DA) for 40 min and rinsed 3 times with phosphate buffer for ROS observation. A universal fluorescence microscope (Olympus BX61, Japan) was used to detect fluorescence. As a control, samples were preincubated with 20 mM DPI (Sigma, D2926) for 20 min. To determine the H_2_O_2_ levels, SOD activity, and CAT activity, the mycelia were incubated in PDB for 3 days and assayed using a kit according to the manufacturer’s instructions (Solarbio, China).

### Localization and determination of free cytoplasmic Ca^2+^

To evaluate the role of ROS in cytoplasmic Ca^2+^ accumulation, OH^−^ was generated by adding a mixture of 2 mM H_2_O_2_, 0.5 mM Cu^2+^, and 0.5 mM ascorbic acid to the mycelia for 30 min after Fluo-4 AM loading (Zhao et al. 2016). A universal fluorescence microscope (Olympus BX61, Japan) was used to detect fluorescence. The concentration of Ca^2+^ in the mycelium was measured using a calcium colorimetric assay kit (Beyotime, S1063S).

### Determination of glutathione/glutathione disulphide (GSH/GSSG) ratio and malondialdehyde (MDA) contents

The mycelium was shaken in PDB for 3–5 days, filtered, dried, and ground with liquid nitrogen. The contents of GSH, GSSG and MDA were determined using GSH/GSSG and MDA assay kits (Beyotime, S0053). All analyses were repeated three times with three replicates each experiment.

### Mitochondrial ultrastructure observation

Mito-Tracker Green was used to examine the distribution of mitochondria. Mito-Tracker Green working solution was added dropwise to the hyphae. The hyphae were then incubated at 37 °C for 30 min, washed three times with PBS and observed under a universal fluorescence microscope (Olympus BX61, Japan).

To visualize the mitochondrial structure, the mycelia were cultured in PDB for 3 days and subsequently fixed with 2.5% glutaraldehyde for 24 h. After slicing and processing the cells, the mitochondria were observed using a transmission electron microscope. Additionally, the Mitochondrial Membrane Potential Assay Kit with TMRE (Tetramethyl rhodamine ethyl ester, Beyotime, C2001S) was utilized to determine the mitochondrial membrane potential.

### Extracellular acid secretion and intracellular OA assays

To detect the secretion of acidic substances, different pH indicators (2 mg/ml methyl red and 1 mg/ml bromophenol blue) were added to the water agar media, and then mycelium block-inoculated branches were placed in contact with the media. The concentration of OA was determined according to the instructions in the oxalic acid content assay kit (Solarbio, BC4365). Briefly, after five days of incubation at 25 °C with shaking at 150 rpm, the mycelia were filtered and dried, and a 0.1 g hyphal sample was weighed for subsequent experiments.

#### Leaf reactive oxygen burst assay

To determine ROS levels in leaves, the poplar leaves were inoculated with mycelium blocks for 24 h, decolorized in 95% ethanol for 3 h, soaked in 1 mg/ml 3,3'-diaminobenzidine (DAB) (Sigma, RES2041D) staining solution for 12 h, and finally observed under an optical microscope.

#### Quantitative real-time PCR (qRT–PCR) analysis

To analyze the transcript levels of NADPH oxidase genes during infection, RNA samples were extracted from dendritic tissues inoculated with wild-type, mutant strains at 0, 12, 24, and 48 h after inoculation (hpi). Each gene assay was performed in biological triplicates, each with three independent technical replicates. Total RNA was isolated with RNA Easy Fast Plant Tissue Kit (TIANGEN, DP452) following the manufacturer’s instructions. RNA was reverse-transcribed using the Quant Script RT Kit (TIANGEN, 4,992,784) for fluorescence-based quantitative PCR experiments. qRT-PCR was performed with Super Real Premix Plus (TIANGE, FP205) using an ABI 7500 real-time PCR system (Applied Biosystems, USA). Each assay was performed in biological triplicate with three independent technical replicates each. The relative expression of genes was calculated by using the 2^−ΔΔCT^ method. All primers were listed in Table S1.

#### Iron carrier secretion assay

Iron carrier release from each strain was detected using Chrome azurol S (CAS) detection solution. The CAS assay solution changes from blue to orange when iron ions in the blue assay plate are seized by the microbial secretion of ferritin. The specific operation is as follows: CAS detection medium (1L: glucose 100g, peptone 20g, magnesium sulfate heptahydrate 0.5g, calcium chloride 0.5g, add deionized water to volume to 800 ml, magnetic stirring to dissolve, autoclaved at 115 °C for 20 min, cooled to 50–60 °C, to the base of the medium, slowly add 60 ℃ preheated 100 ml of 10 × CAS buffer and 100 ml 10 × CAS detection solution were slowly added to the medium base at 60 °C to form solid ligand base containing agar and liquid medium without agar, respectively; Iron carrier release plate observation: inoculate the strain to be tested in CAS medium plate, incubate at 25 °C for 5–7 days, observe whether there is an orange halo around the colony.

#### Statistical analysis

All data are from at least three independent experiments in which each treatment was performed in triplicate. Quantitative data were normalized to 100% for comparison between different treatments. Significance was assessed using SPSS Statistics 22 and Student’s t test. One asterisk indicates a *p* value < 0.05, and two asterisks indicate a *p* value < 0.01.

## Supplementary Information

Additional file 1. Figure S1. Validation of NADPH oxidases deletion mutants and complements in Cytospora chrysosperma.

Additional file 2. Figure S2. CcNox2 affects the uptake and utilization of iron ions in Cytospora chrysosperma.

Additional file 3.

## Data Availability

All data generated or analyzed during this study are included in this published article.

## References

[CR1] Dikalov S (2011) Cross talk between mitochondria and NADPH oxidases. Free Radical Biol Med 2011(51):1289–1301. 10.1016/j.freeradbiomed.2011.06.033 10.1016/j.freeradbiomed.2011.06.033 PMC3163726 21777669

[CR2] Ewald CY, Hourihan JM, Bland MS*, *et al. NADPH oxidase-mediated redox signaling promotes oxidative stress resistance and longevity through memo-1 in *C. elegans*. eLife 2017:6. 10.7554/eLife.19493 10.7554/eLife.19493 PMC5235354 28085666

[CR3] Goswami RS (2012) Targeted Gene Replacement in Fungi Using a Split-Marker Approach. Methods Mol Biol 835:255–269. 10.1007/978-1-61779-501-516 22183659 10.1007/978-1-61779-501-5_16

[CR4] Han Z, Xiong D, Xu Z|*, *et al. The *Cytospora chrysosperma* virulence effector CcCAP1 mainly localizes to the plant nucleus to suppress plant immune responses. mSphere 2021:6. 10.1128/mSphere.00883-20 10.1128/mSphere.00883-20 PMC8544888 33627507

[CR5] Kim HJ, Chen C, Kabbage M et al (2011) Identification and characterization of *Sclerotinia sclerotiorum* NADPH oxidases. Appl Environ Microbiol. 77:7721–7729. 10.1128/aem.05472-11 21890677 10.1128/AEM.05472-11 PMC3209176

[CR6] Lambeth JD (2002) NOX enzymes and the biology of reactive oxygen. Nat Rev Immunol 4:181–189. 10.1038/nri1312 10.1038/nri1312 15039755

[CR7] Lin L, Fan XL, Groenewald JZ, Jami F, Wingfield MJ, Voglmayer H, Jaklitsch W, Castlebury LA, Tian CM, Crous PW. *Cytospora*: an important genus of canker pathogens. Stud Mycol 109: 323–401. 10.3114/sim.2024.109.05 10.3114/sim.2024.109.05 PMC11663427 39717654

[CR8] Liu N, Wang W, He C*, *et al*. *NADPH oxidases play a role in pathogenicity via the regulation of F-Actin organization in *Colletotrichum gloeosporioides*. Front Cellular Infect Microbiol 2022:12. 10.3389/fcimb.2022.845133 10.3389/fcimb.2022.845133 PMC9240266 35782153

[CR9] Malagnac F, Lalucque H, Lepère G et al (2002) Two NADPH oxidase isoforms are required for sexual reproduction and ascospore germination in the filamentous fungus *Podospora anserina*. Fungal Genet Biol. 41:982–997. 10.1016/j.fgb.2004.07.008 10.1016/j.fgb.2004.07.008 15465387

[CR10] Martínez-Reyes I, Diebold, Lauren P, Kong H*, *et al. TCA cycle and mitochondrial membrane potential are necessary for diverse biological functions. Molecular Cell 2016:61:199–209. 10.1016/j.molcel.2015.12.002 10.1016/j.molcel.2015.12.002 PMC4724312 26725009

[CR11] Morita Y, Hyon GS, Hosogi N et al (2012) Appressorium‐localized NADPH oxidase B is essential for aggressiveness and pathogenicity in the host‐specific, toxin‐producing fungus *Alternaria alternata* Japanese pear pathotype. Molecular Plant Pathol. 14:365–378. 10.1111/mpp.12013 10.1111/mpp.12013 PMC6638787 23279187

[CR12] Mu D, Li C, Zhang X et al (2013) Functions of the nicotinamide adenine dinucleotide phosphate oxidase family in *Ganoderma lucidum*: an essential role in ganoderic acid biosynthesis regulation, hyphal branching, fruiting body development, and oxidative‐stress resistance. Environ Microbiol 16:1709–1728. 10.1111/1462-2920.12326 24238263 10.1111/1462-2920.12326

[CR13] Nauseef WM (2008) Biological roles for the NOX family NADPH oxidases. J Biol Chem 283(25):16961–16965. 10.1074/jbc.R700045200 18420576 10.1074/jbc.R700045200 PMC2427363

[CR14] Nordzieke DE, Fernandes TR, El Ghalid M et al (2019) NADPH oxidase regulates chemotropic growth of the fungal pathogen *Fusarium oxysporum* towards the host plant. New Phytol 224:1600–1612. 10.1111/nph.16085 31364172 10.1111/nph.16085

[CR15] Nuhu F, Gordon A, Sturmey R et al (2020) Measurement of glutathione as a tool for oxidative stress studies by high performance liquid chromatography. Molecules 13;25(18):4196. 10.3390/molecules25184196 10.3390/molecules25184196 PMC7571047 32933160

[CR16] Popa CV, Dumitru I, Ruta LL*, *et al*. *Exogenous oxidative stress induces Ca^2+^ release in the yeast Saccharomyces cerevisiae. The FEBS J. 2010:277:4027–4038. 10.1111/j.1742-4658.2010.07794.x 10.1111/j.1742-4658.2010.07794.x 20735472

[CR17] Rana K, Yuan J, Liao H et al (2022) Host-induced gene silencing reveals the role of Sclerotinia *sclerotiorum oxaloacetate* acetylhydrolase gene in fungal oxalic acid accumulation and virulence. Microbiol Res 258:126981. 10.1016/j.micres.2022.126981 35183041 10.1016/j.micres.2022.126981

[CR18] Ryder LS, Dagdas YF, Mentlak TA. et al (2013) NADPH oxidases regulate septin-mediated cytoskeletal remodeling during plant infection by the rice blast fungus. Proceed National Academy Sci 110:3179–3184. 10.1073/pnas.1217470110 10.1073/pnas.1217470110 PMC3581893 23382235

[CR19] Scott B, Eaton CJ (2008) Role of reactive oxygen species in fungal cellular differentiations. Curr opinion microbiol 11(6):488–493. 10.1016/j.mib.2008.10.008 10.1016/j.mib.2008.10.008 18983937

[CR20] Segmüller N, Kokkelink L (2008) Giesbert S*, *et al (2008)NADPH oxidases are involved in differentiation and pathogenicity in *Botrytis cinerea*. Molecular plant-microbe interactions 216:808–819. 10.1094/MPMI-21-6-0808 10.1094/MPMI-21-6-0808 18624644

[CR21] Sharma P, Jha AB, Dubey RS et al (2012) Reactive Oxygen Species, Oxidative damage, and antioxidative defense mechanism in plants under stressful conditions. J Botany 1–26. 10.1155/2012/217037

[CR22] Shen Q, Liang M, Yang F et al (2020) Ferroptosis contributes to developmental cell death in rice blast. New Phytol 227:1831–1846. 10.1111/nph.16636 10.1111/nph.16636 32367535

[CR23] Wang Y, Wang Y (2020) Oxalic acid metabolism contributes to full virulence and pycnidial development in the poplar canker fungus *Cytospora chrysosperma*. Phytopathol 110:1319–1325. 10.1094/phyto-10-19-0381-r 10.1094/PHYTO-10-19-0381-R 32154765

[CR24] Wang F, Gao W, Sun J, et al (2020) NADPH Oxidase ClNOX2 regulates melanin-mediated development and virulence in Curvularia lunata. Molecular Plant-Microbe Interact 33:1315–1329. 10.1094/mpmi-06-20-0138-r 10.1094/MPMI-06-20-0138-R 32815478

[CR25] Wang W, Wang S, Gong W*, *et al (2022)*Valsa mali* secretes an effector protein VmEP1 to target a K homology domain-containing protein for virulence in apple. Mol Plant Pathol 23:1577–1591. 10.1111/mpp.13248 10.1111/mpp.13248 PMC9562843 35851537

[CR26] Wu Y, Xu L, Yin Z et al (2018) Two members of the velvet family, VmVeA and VmVelB, affect conidiation, virulence and pectinase expression in *Valsa mali*. Mol Plant Pathol 19:1639–1651. 10.1111/mpp.12645 10.1111/mpp.12645 PMC6638101 29127722

[CR27] Wu, P. C., Chen, C. W., Choo, C. Y. L.*, et al.*,(2020).Biotin biosynthesis affected by the NADPH oxidase and lipid metabolism is required for growth, sporulation and infectivity in the citrus fungal pathogen *Alternaria alternata*. Microbiol Res 241:126566.10.1016/j.micres.2020.126566 10.1016/j.micres.2020.126566 33032167

[CR28] Xie D, Tang C, Wang Y, Jin H, Wang Y (2023) The Transcription Factor CcRlm1 Regulates Cell Wall Maintenance and Poplar Defense Response by Directly Targeting *CcChs6* and *CcGna1* in Cytospora chrysosperma. Appl Environ Microbiol 89:e00661-e723. 10.1128/aem.00661-23 37289076 10.1128/aem.00661-23 PMC10304682

[CR29] Xiong D, Yu L, Shan H et al (2021) CcPmk1 is a regulator of pathogenicity in *Cytospora chrysosperma* and can be used as a potential target for disease control. Molecular Plant Pathology 22:710–726. 10.1111/mpp.13059 10.1111/mpp.13059 PMC8126189 33835616

[CR30] Xu L, Liu H, Zhu S*, *et al (2023) VmPacC-mediated pH regulation of *Valsa mali* confers to host acidification identified by comparative proteomics analysis. Stress Biol 3:18. 10.1007/s44154-023-00097-y 10.1007/s44154-023-00097-y PMC10441875 37676527

[CR31] Yang SL, Chung KR (2012) The NADPH oxidase-mediated production of hydrogen peroxide (H2O2) and resistance to oxidative stress in the necrotrophic pathogen *Alternaria alternata* of citrus. Molecular Plant Pathol 13:900–914. 10.1111/j.1364-3703.2012.00799.x 10.1111/j.1364-3703.2012.00799.x PMC6638813 22435666

[CR32] Yang SL, Chung KR (2013) Similar and distinct roles of NADPH oxidase components in the tangerine pathotype of *Alternaria alternata*. Molecular Plant Pathol 14:543–556. 10.1111/mpp.12026 10.1111/mpp.12026 PMC6638896 23527595

[CR33] Zhang M, Xie S, Zhao Y et al (2019) Hce2 domain-containing effectors contribute to the full virulence of *Valsa mali* in a redundant manner. Mol Plant Pathol 20:843–856. 10.1111/mpp.12796 30912612 10.1111/mpp.12796 PMC6637899

[CR34] Zhang X, Zong Y, Gong D et al (2021) NADPH Oxidase regulates the growth and pathogenicity of *Penicillium expansum*. Front Plant Sci 12. 10.3389/fpls.2021.696210 10.3389/fpls.2021.696210 PMC8387719 34456938

[CR35] Zhang Z, Huang Q, Zhao D et al (2023) The impact of oxidative stress-induced mitochondrial dysfunction on diabetic microvascular complications. Front Endocrinol 14. 10.3389/fendo.2023.1112363 10.3389/fendo.2023.1112363 PMC9941188 36824356

[CR36] Zhao YL, Zhou TT, Guo HS (2016) Hyphopodium-specific VdNoxB/VdPls1-dependent ROS-Ca^2+^ signaling is required for plant infection by *Verticillium dahliae*. PLoS Pathogens 12:e1005793. 10.1371/journal.ppat.1005793 27463643 10.1371/journal.ppat.1005793 PMC4962994

[CR37] Zhao H, Ding X, Chu X et al (2023) Plant immune inducer ZNC promotes rutin accumulation and enhances resistance to *Botrytis cinerea* in tomato. Stress Biology 3:36. 10.1007/s44154-023-00106-0 37676331 10.1007/s44154-023-00106-0 PMC10444710

