# Supplementary figures and images for: Roles of NADPH oxidases in regulating redox homeostasis and pathogenesis of the poplar canker fungus Cytospora chrysosperma
Source: Stress Biol. 2025 May 8;5(1):33. doi: 10.1007/s44154-025-00223-y (PMC12061831; doi:10.1007/s44154-025-00223-y)

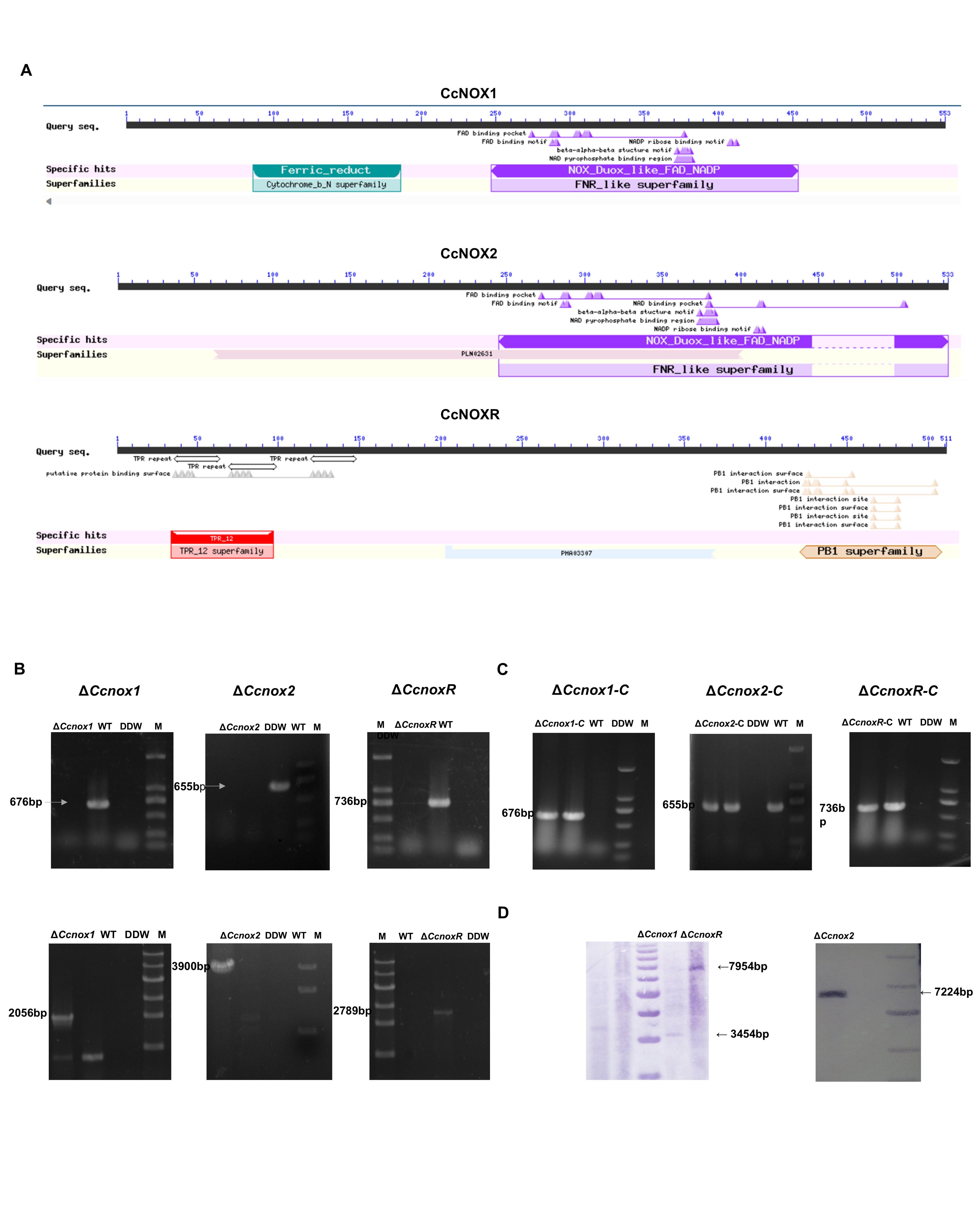

Supplement: Supplementary file 1 — Additional file 1. Figure S1. Validation of NADPH oxidases deletion mutants and complements in Cytospora chrysosperma. [file 44154_2025_223_MOESM1_ESM.jpg]

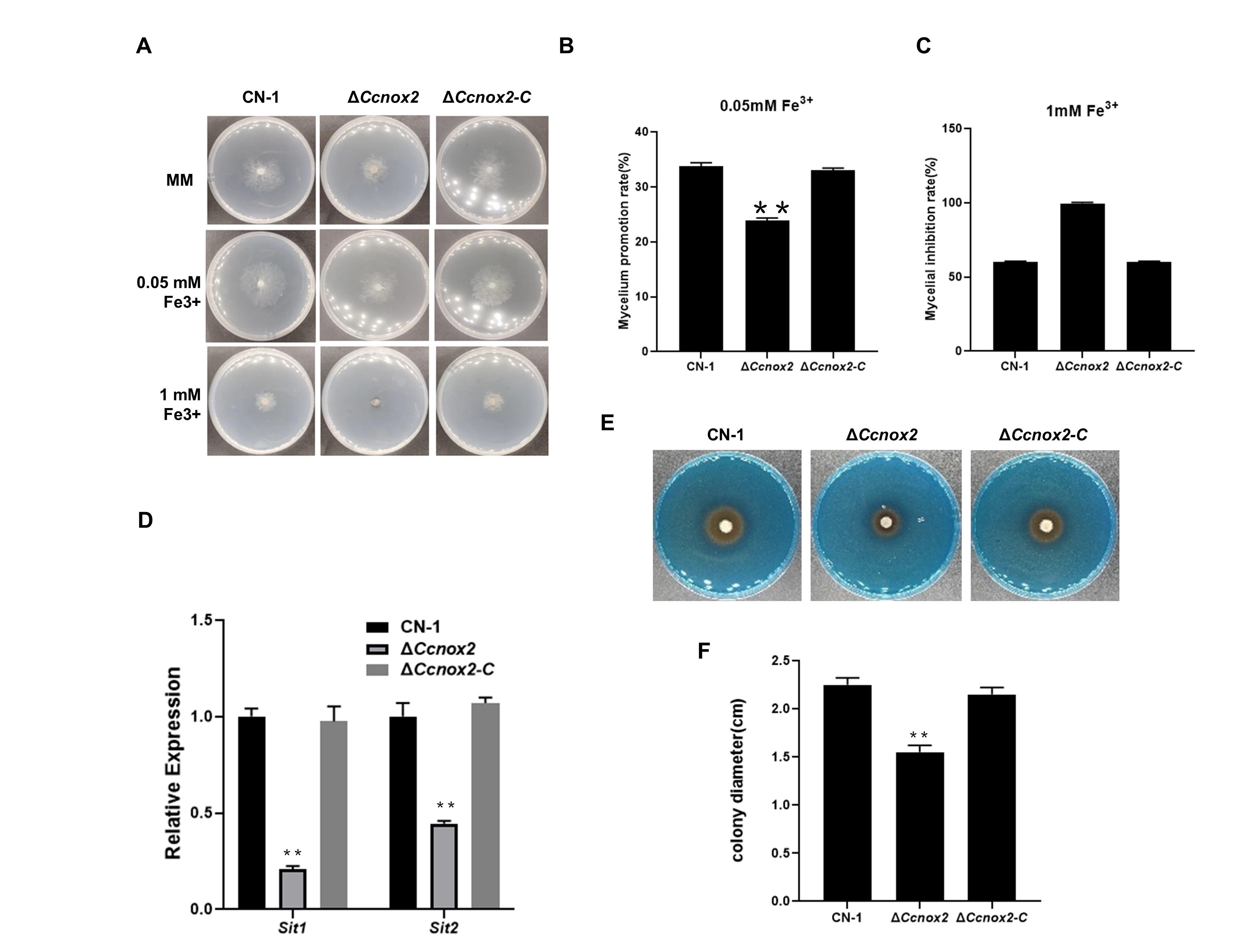

Supplement: Supplementary file 2 — Additional file 2. Figure S2. CcNox2 affects the uptake and utilization of iron ions in Cytospora chrysosperma. [file 44154_2025_223_MOESM2_ESM.jpg]
